# A 10-step participatory program for developing, implementing, and evaluating physical activity promoting actions in nursing homes in Germany

**DOI:** 10.1186/s12889-024-17727-3

**Published:** 2024-02-09

**Authors:** Lea-Sofie Hahn, Ansgar Thiel, Viola Dembeck, Daniel Haigis, Leon Matting, Rebekka Pomiersky, Gerhard W. Eschweiler, Andreas M. Nieß, Gorden Sudeck, Annika Frahsa

**Affiliations:** 1 Institute of Sports Science, University of Tübingen, Tübingen, Germany; 2 Department of Sports Medicine, University Hospital of Tübingen, Tübingen, Germany; 3 Geriatric Centre, University Hospital of Tübingen, Tübingen, Germany; 4 Institute of Social and Preventive Medicine, University of Bern, Bern, Switzerland; 5 Interfaculty Research Institute for Sport and Physical Activity, University of Tübingen, Tübingen, Germany

**Keywords:** Physical Activity Promotion, Nursing homes, Participatory Integrated Counselling Approach

## Abstract

**Background:**

Since multidimensional barriers challenge nursing homes, a socio-ecological approach is needed for physical activity promotion in this setting. So far, little is known about how such an approach can be transferred into the successful development and implementation of PA-promoting actions together with stakeholders on-site. We aimed to investigate the actions and dimensions of PA-promoting actions and their sustainable implementation. To contribute to closing this gap, we present a 10-step program for co-developing and co-evaluating PA-promoting actions in nursing homes through an integrated counselling approach.

**Methods:**

We used a multiple case study approach that built upon manifold data sources, collected in 7 nursing homes over 3 years between 2021 and 2023. We collected fieldnotes and photologs from 14 future workshops (2 per home); 7 evaluation workshops (1 per home); 36 individual counsellings (2 sessions per resident), as well as 87 implementation protocols (action type and frequency), 11 evaluation questionnaires (changes among resources, cooperations, and collaborations); 7 goal attainment scales and 18 individual activity schedules. In addition, we retrieved and documented progress information at regular intervals by phone or email.

**Results:**

With staff, residents, relatives, and volunteers, we co-developed 112 ideas for PA promotion; from which 54 ideas were implemented and integrated into everyday life, differentiated into “activities of daily living,” “structured activities,” and “activity-friendly environments.”; 18 residents in 4 homes participated in individual counselling to develop individual activity schedules. Eighteen actions were rated as “(much) more successful than expected”; 10 “(much) worse than expected,” and 23 “as successful as expected.” Three actions were not evaluated.

**Discussion:**

The participatory integrated counselling approach led to home-specific actions and promoted implementation into everyday life. The number and dimensions of actions implemented largely depended on the mission and vision of the respective home. The lack of staff could partially be compensated for by involving neighbourhoods, volunteers, and community organisations, such as local clubs.

**Conclusion:**

To effectively promote PA in nursing homes, a tailored approach considering structural conditions, locations, volunteer engagement, and organisational visions is essential. Long-lasting partnerships and low-threshold opportunities prove promising. Future research should delve into structural-level change processes and outcomes in this context.

## Introduction

Regular sufficient physical activity (PA) can improve nursing home resident physical and mental performance and health as well as their quality of life and social interactions [1–4]. Sedentary behaviour poses an enormous health risk and even low-intensity activities, such as gardening, can increase independence and individual mobility [5, 6]. At the same time, PA promotion in nursing homes tends to be hindered by multidimensional barriers on several levels.

Socio-ecological models provide a framework for analysing and developing actions to overcome existing barriers [4, 7, 8]. According to Sallis et al. (2006), PA occurs in *behavioural settings*, such as neighbourhoods, which are especially important for physically active residents and people who visit. Inside nursing homes, several DOMAINS OF ACTIVE LIVING represent areas of potential PA (active recreation, household activities, occupational activities, and active transport) affected by several environments. *Perceived environments* are decisive factors for nursing home residents choosing whether they participate in interventions and how comfortable they feel during PA [7]. The availability, accessibility, and safety of indoor and outdoor areas significantly impact resident PA, especially for mobile residents [9, 10]. Inadequate indoor building infrastructure [11–13], unsuitable premises [14], or bad weather conditions like heatwaves [15] also count as environmental barriers and negatively influence and challenge PA promotion. *Policy environments* concern homes (e.g., missions and visions), carriers (e.g., PA promotion embedded in concepts), or governments (e.g., legal requirements and guidelines) [7]. Political decisions, such as restrictions taken during health-threatening events, especially decrease the amount of resident PA [16]. Other potential influences on PA promotion can be found in *information-* (e.g., communication structures), *social cultural-* (e.g., social climate), and *natural*-*environments* (e.g., weather) [7]. Moreover, *intrapersonal conditions* influence individual PA behaviour, especially for advancing age since changes occur day to day [7]. Since residents tend to have various physical and/ or cognitive impairments [2, 11, 17, 18], tailored PA programs are beneficial from health perspectives, increase resident motivation to participate, and enhance their knowledge about advantages of sufficient PA [9, 11, 14, 18, 19]. Particularly residents with predominantly sedentary lifestyles before moving into nursing homes benefit from tailored programs [19]. However, nursing staff often feel insufficiently trained or unmotivated to provide structured PA programs, afraid of injuring themselves or residents [2, 18]. This phenomenon is often related to a lack of support from home management or carriers [2, 11, 14, 18] because PA promotion is deprioritised by facilities or rigid organisational structures make it difficult to integrate sufficient PA into everyday life [16]. Meals, basic care, and other fixed components of home routines often leave no room for PA promotion, which particularly affects immobile residents unable to go for strolls or perform other small activities independently [9, 11, 14, 18, 20].

Despite vast knowledge about PA benefits for older adults, little research exists about implementing PA-related actions in nursing home settings [21–27]. Most studies address intrapersonal conditions, particularly for residents with cognitive impairments, such as dementia, to reduce risks of fall [21–23] or slow cognitive decline [24]. Other studies aim to increase quality of life and decrease depressive symptoms [25, 26]. Furthermore, studies on intrapersonal levels investigate effects of individually tailored intervention programs [27] or aim to improve future PA programs or activities of daily living through their investigations [1, 28]. Other studies consider home-policy environments, such as embedding PA programs into nursing home daily routines, yet do not consider perceived environments [29, 30]. Only a few studies consider intrapersonal conditions and the social cultural, home-policy, and perceived environment at the same time [31–33]. Moreover, these studies either include only specific groups of residents [33] or involve only occupational therapists and not nursing staff [32]. Many studies apply predetermined, time-limited interventions in different homes and do not take individual contexts into account [21, 24, 25]. Only a single study identified the need for a flexible, inclusive approach to improve resident PA and the necessity for staff to communicate PA benefits and requirements to residents [31].

Less is known about how a process can look like that successfully develops and implements PA-promoting actions together with nursing home staff and different relevant actor groups, such as relatives, external activity promoters, and volunteers. Furthermore, the increasing numbers of migrants working in care affect the understanding of gender and cultural diversity and also affect everyday life in nursing homes in coming decades [4]. Thus, considering intrapersonal conditions and different environments is crucial when it comes to PA promotion in nursing homes. In response, we present a 10-step program for developing and evaluating PA-promoting actions in nursing homes working with staff, residents, relatives, and volunteers within a participatory integrated counselling approach. We consider all levels of the socio-ecological model [7] to sustainably implement PA-promoting actions into everyday nursing home life. We investigate the development, implementation and evaluation of PA-promoting actions that aim to change everyday activity and PA-related structures of the nursing home.

## Materials and methods

Our study took place within the larger *BaSAlt* project on PA promotion and counselling in nursing homes (German Federal Ministry of Health 2019–2023, grant no. ZMVI1-2519FSB114). Homes of four different non-profit carriers in the Federal State of Baden-Wuerttemberg, Germany, were chosen to represent different forms of organisations regarding environmental contexts (periphery/urban), capacity (33–52 living places), and resident population composition [34]. More women than men lived in all 7 homes; 2 homes included protected areas for residents with dementia—this is the reason why these 2 homes had more residents with dementia compared to the other homes.

We used a multiple case study approach to examine real-world, contemporary, multiple-bounded systems (cases) with longitudinal detailed, in-depth data collection. We built upon manifold sources of information, including ethnographic field notes and documents collected over 3 years between 2021 and 2023 [35]. We conducted organisational counselling, including 21 workshops, with people living and working in this setting and individual counselling with 18 residents and their relatives. We conducted the participatory integrated counselling approach in 7 homes (an eighth home dropped out due to the pandemic). We collected fieldnotes and photologs from 14 future workshops (2 per home); 7 evaluation workshops (1 per home); and 36 individual counsellings (2 sessions per resident), as well as 87 implementation protocols (action type and frequency); 11 evaluation questionnaires (changes among resources, cooperations, and collaborations); 7 goal attainment scales (GAS) [36]; and 18 individual activity schedules. In addition, we retrieved and documented progress information at regular intervals by phone or email.

We used a participatory integrated counselling approach to promote PA in nursing homes. Organisational counselling was based on 3 workshops (future workshop I + II and an evaluation workshop) and 2 individual counselling sessions (individual counselling I + II). We developed and used a 10-step program (Table 1) to plan and implement PA-promoting actions and assess goal attainment. Scientific project team members guided all workshops and individual counselling. The 7 nursing homes received €8500 each to put PA-promoting actions—developed from participatory integrated counselling—into practice. Ideally, individual counselling I starts directly after future workshop I; individual counselling II takes place 6 weeks after individual counselling I; and evaluation workshop occurs 6 months after future workshop II to guarantee sufficient time to implement PA-promoting actions into everyday life.

**Table 1:** 10-step program

**FUTURE WORKSHOP I**
Step 1 – Collecting potential actions & discussing in small groups Following a brainstorming session about potential PA-promoting offers with all participants, small groups discussed favoured actions together with researchers to consider different perspectives.
Step 2 – Cataloguing actions Actions were catalogued and organised by *opportunities*, *resources*, *commitments*, and *goals* for promoting PA.
Step 3 – Favouring & considering actions in detail As homework, participants collected actions they wanted to implement in daily home routine and considered actions in detail to present to the expert team.
**FUTURE WORKSHOP II**
Step 4 – Planning actions Actions prioritised were systematically planned following the SMART concept [37] – including specification, measurability, acceptance, realisability and timing (further details below).
Step 5 – Creating goal attainment scaling 1.0 To assess success later, each action was assessed according to GAS [36]. Therefore, participants set expected goals by phrasing action success in 1 sentence (GAS = 0). The expert team determined positive and negative gradations (GAS = -2, -1, + 1, +2) retrospectively using field notes from workshops, then submitted them to homes for confirmation. Additionally, participants decided whether individual counselling for residents should be offered by a team of experts in their homes within the next weeks.
**INDIVIDUAL COUNSELLING I + II**
Step 6 – Developing individual activity schedules Individual counselling I was structured according to the 5 A concept [38] (further details below) and aimed to integrate PA opportunities, including actions developed in future workshops, into individual daily resident lives based on individual PA motives and goals [39]. Relatives supported cognitively impaired residents during individual counselling and in implementing the activity schedule.
Step 7 – Handling barriers After 6 weeks, individual counselling II was scheduled with residents and relatives to handle possibly arising barriers and to reflect activities scheduled together.
**EVALUATION WORKSHOP**
Step 8 – Implementing-supervising Implementing actions into everyday life was supervised by the project team over 6 months between future workshops I + II by providing support when problems arose. Within 6 months, homes integrated actions into organisational structures and daily routines [40]. Responsible persons sent implementation protocols regularly. Evaluation questionnaires were surveyed after 3 and 6 months.
Step 9 – Revising goal attainment scaling 2.0 In the evaluation workshop, actions developed during future workshop I + II were evaluated according to a 5-level GAS. Actions were rated in different areas (social, neighbourhood, green care, infrastructure, employees & caregivers, individual activity behaviour, and specifications of the nursing home) based on the GAS of future workshop II. To design the GAS lower threshold, rating scales were reformulated into statements (-2 = much worse than expected; -1 = worse than expected; 0 = as expected; +1 = better than expected; +2 = much better than expected).
Step 10– Goal Attainment Scaling 3.0 Individual counselling was rated according to GAS, as well evaluated the success of the 2 sessions. Success was not evaluated for each resident but for the counselling approach in the home.

In Future Workshop II, actions were systematically planned according to the SMART concept (Table 2) [37]. The actions were described (**S**pecification), success was defined (GAS = 0) (**M**easurability), and single actions were voted on in plenary (**A**cceptance). If there was a simple majority, the action was approved for implementation into everyday life. Necessary preparations for implementation were collected and responsibilities allocated (**R**ealisability). Lastly, a start date was set (**T**iming).

**Table 2:** Steps of the SMART-concept

Specification	Describing the action
**M**easurability	Defining success
**A**cceptance	Consenting the team
**R**ealisability	Planning implementation
**T**iming	Starting the action

Individual counselling I + II followed the 5 A concept [38] (Table 3). First, in individual counselling I, individual motives and goals for PA were identified together with residents and relatives. Personal requirements were considered as well as socio-infrastructural conditions (**A**ssess). Second, experts (e.g., project team or physiotherapists) made recommendations for PA – based on the needs and requirements identified (**A**dvise). Third, preferred activities were recorded in an individualised activity schedule, and goals were defined (**A**gree). Fourth, if desired, experts provided support to make successful implementation more likely (e.g., coping plans) (**A**ssist). After 6 weeks, possible barriers to implementation were discussed in individual counselling II and, if possible, eliminated (**A**rrange).

**Table 3:** Steps of the 5 A-concept

**A**ssess	Identifying personal activity goals [39] and considering individual needs and nursing home social-infrastructural conditions
**A**dvise	Advising from PA experts about needs
**A**gree	Agreeing upon goals by recording individual activities in resident activity schedules
**A**ssist	Providing support for changing behaviour, such as action or coping plans)
**A**rrange	Arranging a follow-up meeting 6 weeks later to handle barriers

## Results

We aimed to investigate the actions and dimensions of PA-promoting actions developed during our participatory integrated counselling approach as well as their sustainable implementation in the structures of the nursing home. The analytic approach was situated within the socio-ecological framework [7], which allowed us to consider the complexity of promoting PA in the nursing home setting. We present results regarding (1) actions and dimensions of PA promotion implemented in participating nursing homes—developed in future workshop I + II and individual counselling I + II—followed by (2) GAS for evaluating implementation [36].

### PA-related actions and dimensions in participating nursing homes

A total of 112 potential actions for promoting PA were developed in the 7 nursing homes during future workshop I. The number of actions varied between nursing homes, ranging from 12 to 20. The nursing homes selected 50% of the potential actions (*n* = 57) for further development in future workshop II, then implemented into everyday life. During individual counselling I + II, actions from future workshops were integrated into weekly resident schedules (Monday–Friday), as well as therapy appointments and individual PA opportunities, such as strolls with relatives. The participatory approach allowed everyone to contribute as many ideas for potential actions as possible using a brainstorming method. In a second step, the results of the brainstorming process were prioritised considering the feasibility of the potential actions.

**Fig. 1:**
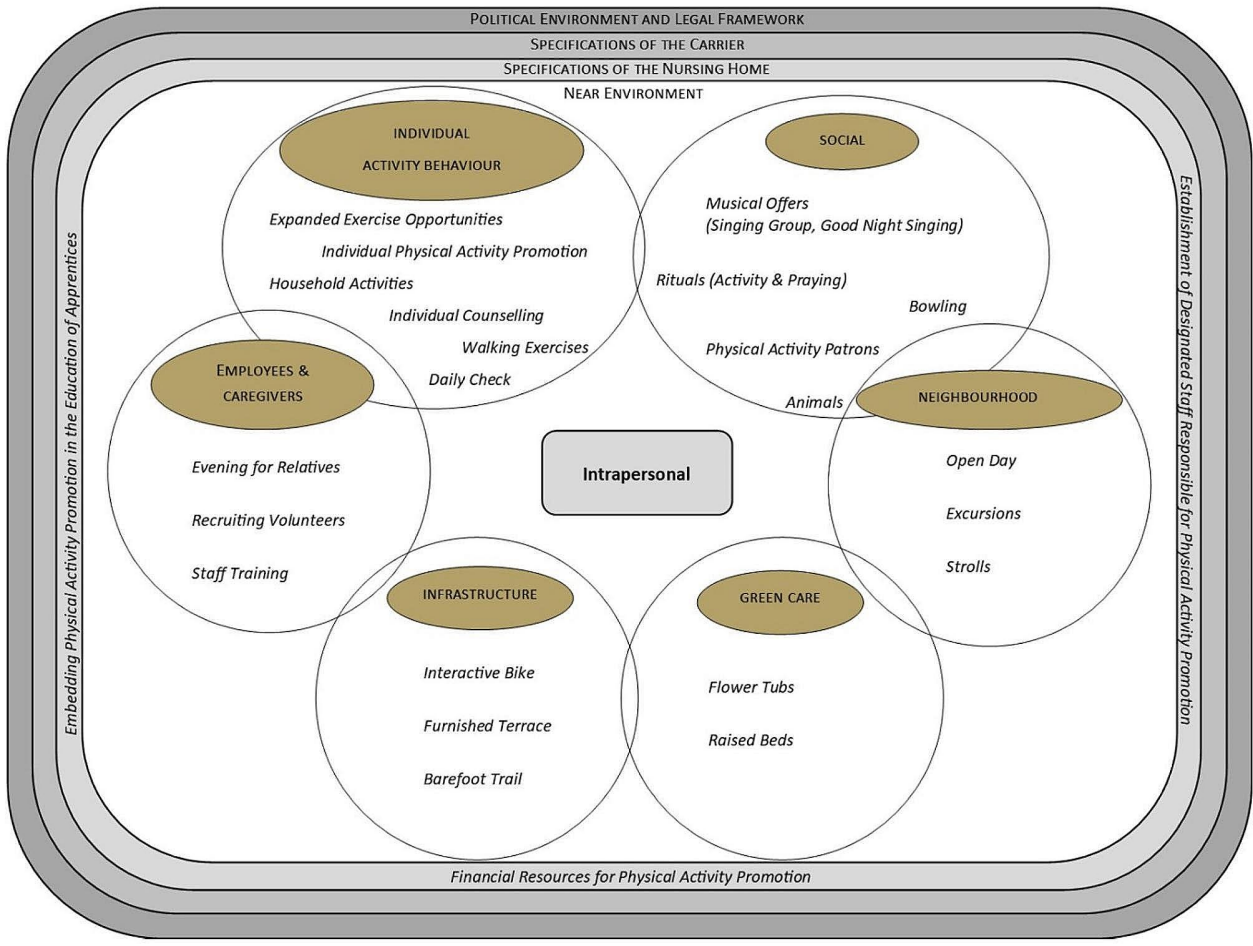
Areas of physical activity promotion

​According to Sallis et al.’s [7] and Bauman et al.’s [8] socio-ecological models, Fig. 1 presents the PA-promoting actions within different environments. The inner circle represents the near environment and *Related Actions*. The outer circle represents external influencing factors on PA and *Supporting Actions* for areas in the inner circle.

Three external factors influenced PA promotion in the outer circle: Political Environment and Legal Framework (e.g., laws and regulations in care related to PA promotion); Specifications of the Carrier (e.g., mission statement); and Specifications of the Nursing Home (e.g., home’s vision and mission statement, job descriptions, or budget planning). The management level of nursing homes also provided supporting actions to integrate PA promotion into organisational structures. Three supporting actions developed in the counselling approach included the *Establishment of Designated Staff Responsible for PA Promotion* (*n* = 7 implementations); *Financial Resources for PA Promotion* (*n* = 7 implementations); and *Embedding PA Promotion in the Education of Apprentices* (*n* = 1 implementation). All external influencing factors promoted or hindered PA in nursing homes.

Six PA programs to increase individual activity behaviour were represented the most in the inner circle. Four out of seven homes made use of *Individual Counselling* to identify individual activity motives of 18 residents and make their everyday lives more active according to their interests. The opportunity to participate in *Household Activities*, such as baking or folding laundry was offered in every nursing home. A *Daily Check* was implemented in 2 homes and aimed to scan everyday life at regular intervals to identify potentials for low-threshold PA opportunities. Five social activities within the homes were a popular way of indirectly promoting PA, such as *Musical Offers* and cooperating with external activity promoters (*Animals or PA Patrons*). However, 3 PA-promoting actions such as *Strolls*, or short *Excursions* also extended beyond boundaries of homes into the neighbourhood. A popular activity among older people was green care with *Raised Beds* or *Flower Tubs*. There was also a focus on employees & caregivers in 5 homes to provide highly qualitative activities for residents. Staff received *Training* to expand their PA offers and expertise. In addition to *Recruiting Volunteers*, family members were also made aware of PA (*Evening for Relatives*). Three actions were also implemented in infrastructure. First, an *Interactive Bike* designed to motivate residents by showing videos of routes they cycled. Second, a *Barefoot Trail* for mobile residents and wheelchair users. Third, a *Furnished Terrace* with chairs, banks, and umbrellas to create attractive meeting places, residents enjoyed going to.

In summary, the number of actions varied greatly from home to home (ranging from 5 to 12). We adapted the 4 DOMAINS OF ACTIVE LIVING [7] for the special conditions of nursing home settings. Table 4 depicts the classification of the developed PA-promoting actions implemented in the near environment of the nursing homes and either directly or indirectly promoted PA.

**Table 4:** Classification of the developed actions into adapted DOMAINS OF ACTIVE LIVING according to Sallis et al. (2006)

Activities of Daily Living	Structured Activities	Activity-Friendly Environment
*Directly* • Flower Tubs • Raised Beds • Household Activities *Indirectly* • Daily Check	*Directly* • Musical Offers • Rituals • Bowling • Individual PA Promotion *Indirectly* • Expanded Exercise Opportunities • Individual Counselling • Evening for Relatives • Recruiting Volunteers Staff Training	*Directly* • Walking Exercises • Excursions • Strolls • Interactive Bike • Barefoot Trail *Indirectly* • PA Patrons • Animals • Open Day • Furnished Terrace

### Success of implemented PA-promoting actions

Of the 57 total actions selected—developed during future workshop I + II—54 (95%) were implemented in different areas. Eighteen actions (33%) were rated as “(much) more successful than expected”; 23 (43%) “successful as expected”; 10 (19%) “(much) worse than expected”; and 3 actions could not be evaluated (Table 5).

**Table 5:** Results of the goal attainment scaling split into physical activity-promoting areas

	Much worse than expected	Worse than expected	As successful as expected	More successful than expected	Much more successful than expected	Not evaluated
social	3		1	1	3	
neighbourhood	1			4	1	1
green care	1		3	1	1	
infrastructure			3			
employees & caregivers	1				1	2
individual activity behaviour	1		4	3	3	
specification of the nursing home		3	12			
**Total**	7	3	23	9	9	3

*Financial Resources* and *Establishment of Designated Staff Responsible for PA Promotion* were assessed as “successful as expected” in all nursing homes (specifications of the nursing home). A *Daily Check* for low-threshold PA-promoting potentials was successfully realised in 1 of 2 homes and *Individual Counselling* for residents was conducted in 4 of 7 nursing homes (individual activity behaviour). Overall, almost all actions for increasing individual activity behaviour were rated “successful as expected” or better (10 out of 11 actions). *Strolls* (supervised and unsupervised) and cooperating with external activity promoters (*Animals* or *PA Patrons*) were frequently applied actions within neighbourhood and social. Actions were evaluated as “much more successful than expected” in all performing homes as well as a large number of *Musical Offers* (social). However, participating nursing homes evaluated social actions heterogeneously. Actions directed at employees & caregivers were implemented successfully into the nursing home structures, except for *Recruiting Volunteers*. green care actions were rated mostly “as successful as expected.”

A glance at the individual homes shows that 5 of 7 that participated in workshops rated success negatively and positively (Table 6). In 2 homes, only positive evaluations were achieved. Whether homes were located in urban or periphery areas did not make a difference.

**Table 6:** Goal attainment scaling divided by home

	Much worse than expected	Worse than expected	As successful as expected	More successful than expected	Much more successful than expected	Not evaluated
**Home 1** (periphery)	1	1	4	2		2
**Home 2** (periphery)			3	1	2	
**Home 3** (periphery)			4	1		
**Home 4** (urban)	1		6			
**Home 5** (periphery)	1	1	1	3	2	
**Home 6** (urban)	Dropped Out					
**Home 7** (urban)	1		3		2	
**Home 8** (periphery)	3	1	2	2	3	1
**In total**	**7**	**3**	**23**	**9**	**9**	**3**

*Individual Counselling* was conducted in 4 of 7 nursing homes with participant numbers ranging from 2 to 6 (individual activity behaviour). Success was assessed for all counselling overall in each home (Table 7). Results were mostly positive; only 1 nursing home rated *Individual Counselling* “much worse than expected,” which was due to a low number of participants.

**Table 7:** Success evaluation of the individual counselling

	Much worse than expected	Worse than expected	As successful as expected	More successful than expected	Much more successful than expected	Number of residents with individual counselling
**Home 1** (periphery)			x			6
**Home 2** (periphery)	no individual counselling					
**Home 3** (periphery)	no individual counselling					
**Home 4** (urban)	x					2
**Home 5** (periphery)				x		6
**Home 6** (urban)	dropped out					
**Home 7** (urban)					x	4
**Home 8** (periphery)	no individual counselling					
**Total**						**18**

## Discussion

We developed a 10-step program for developing, implementing, and evaluating PA-promoting actions in nursing homes by using a participatory integrated counselling approach. We now discuss 3 principal findings: 1) the participatory integrated counselling approach leads to a wide range of home-specific actions; (2) the need for adapting the DOMAINS OF ACTIVE LIVING to cover the special conditions of the setting and specific nursing homes; and (3) the relevance of cooperation when it comes to PA promotion.

### Addressed areas of PA promotion when using a participatory integrated counselling approach

When it comes to PA promotion, nursing homes are facilities with special conditions obligated to provide offers for maintaining and promoting mobility [41]. They are individually different from their structural conditions, peripheral or urban locations, the number of volunteers, and the mission concept of the carrier (specification of the nursing home). In addition, barriers often exist for PA promotion at environmental [2, 9, 11, 14, 17, 18]; individual [2, 9, 11, 14, 17–20]; and organisational [2, 9, 11, 14, 18, 20] levels requiring development of home-specific actions. We used a participatory integrated counselling approach making it possible to consider different intrapersonal, socio-cultural, organisational, environmental, and political prerequisites and thus developing home-specific actions for more active everyday lives [7, 8] (Fig. 1). Using a participatory integrated counselling approach means active involvement when developing actions and supporting implementation into nursing home everyday life. Actions are adapted for residents who are frail, cognitively impaired, or with dementia and their benefit [2, 11, 17, 18], and the probability of reaching residents with previously sedentary lifestyles increases [19]. *Staff Training* (employees & caregivers) and resulting *Expanded Exercise Opportunities* (individual activity behaviour) empowered staff to feel competent to provide PA-promoting actions [2, 18], which also resulted in staff communicating benefits of sufficient PA to residents and relatives, increasing their motivation and participation [9, 14, 18, 19]. In all participating homes during different workshops, we connected people with otherwise little or no opportunities for exchange and communication on these topics due to their working positions. Sallis et al. (2006) and Sauter et al. (2019) already confirmed the importance of information sharing and communication structures. For example, regular visits from a therapy dog required cooperation from many parties, such as home management, volunteers, and external activity promoters. It turned out the dog provided more than therapy only; the dog promoted PA on 4 legs that could increase quality of life and social interactions [1–4]. When the therapy dog visited the home, residents left their rooms to see and play with the dog (social). Implementing actions—in general—was only possible with home management support [2, 11, 14, 18], which provided *Responsible Staff* as well as *Financial Resources for PA Promotion* (specification of the nursing home). By prioritising PA promotion and thus embedding actions in weekly and annual plans, activating residents was possible even within rigid organisational structures [9, 11, 14, 18, 20].

### Assigning PA-promoting actions in DOMAINS OF ACTIVE LIVING

PA as a multidimensional construct requires a socio-ecological approach to adequately capture its complexity. We drew on Sallis’ model [7] since all areas of activity are covered at different levels. We adapted the DOMAINS OF ACTIVE LIVING to the nursing home setting to better illustrate the special conditions of the setting. Nursing homes vary greatly concerning their structural conditions, peripheral or urban locations, number of volunteers, or carrier missions and visions [16, 42]. Residents with physical or mental disabilities depend on protected environments that only partially reflect their former everyday lives. DOMAINS OF ACTIVE LIVING include (1) Activities of Daily Living; (2) Structured Activities; and (3) Activity-Friendly Environment.

### Activities of daily living

Activities of daily living are among the most effective opportunities to activate residents. Through biography work, earlier interests are queried and considered. Popular activities of daily living in nursing homes are low-intensity activities, such as setting the table, doing laundry, or baking [5, 6]. These activities are intended to provide self-occupation and diversion, and residents feel valued and needed [25, 26]. Activities of daily living are meaningful for residents and do not require great amounts of material and time or financial and personnel resources.

Due to high sedentariness in this setting, even the slightest activities of daily living, like table setting or distributing newspapers at tables, were successful activations. In this regard, it was essential to ask about earlier interests when moving in to fulfil individual preferences. Activities of daily living could be integrated successfully into daily home routines without great expenditures of time, personnel, or money [7]. For example, challenges during the pandemic involved hygiene regulations that prohibited working with food, like peeling potatoes, or folding laundry. Yet meaningful activities lead to physical and mental improvements for residents and create feelings of engagement, independence, and value [1, 2, 7].

### Structured activities

Nursing homes should create a balanced mix of activating and regenerating activities. In contrast to the activities of daily living, which are used by residents to pass time, structured activities should intend to be functional and also cover specific preventive (e.g., fall prevention) as well as therapeutic aspects [21–26]. They can be directly (e.g., strolls) or indirectly (e.g., singing) active, yet always require a guiding person (e.g., external activity promoter or nursing staff).

In our project, each nursing home possessed special characteristics reflected in individual actions and covered the adapted DOMAINS OF ACTIVE LIVING to different extents. In total, 54 PA-promoting actions were integrated into the everyday nursing home life. Implementation was strongly modulated by home missions and visions [7], which—in contrast to Sallis et al.—showed a greater impact than carrier specifications. One home developed almost entirely directly structured activities implemented in weekly schedules. Another home sought to promote PA by offering social events that motivated residents to leave their rooms and meet one another (indirectly structured activities). Both homes belonged to the same carrier.

### Activity-friendly environment

Due to physical and cognitive limitations, most residents are unable to leave and move around nursing homes independently [2, 11, 17, 18]. Thus, it is even more important to provide adequate infrastructures (e.g., walking aids or handrails) and opportunities (e.g., PA-promoting objects) for PA inside the nursing homes [11–13]. Infrastructures outside nursing homes primarily include visits from relatives or friends, but also public and private transportation for external activity promoters, such as therapists or volunteers. For the few residents who still leave nursing homes independently, the walkability (supermarkets, bakeries, or parks for strolling within walking distance) modulates PA positively [7]. Cooperations with clubs or institutions in nearby environments are especially beneficial for immobile residents since they cannot leave homes without accompaniment.

Infrastructural conditions varied greatly from home to home [11–13] and aisle widths or balconies could not be changed easily. But actions like a *Furnished Terrace*, *Barefoot Trail*, *Flower Tubs*, or *Raised Beds* led to an activity-friendly environments within nursing home boundaries and guaranteed fresh air activities or regeneration [7]. Another popular action was supervised *Strolling*, which generated relatively high personnel costs. An activity-friendly environment could save staff resources by allowing cognitively and physically fit residents to go for walks unsupervised or take walks in the home’s garden rather than in neighbourhoods. The advantages of safe and attractively designed outdoor areas in terms of PA became apparent again [7]. The definition of the word *Stroll* was also crucial. If a *Stroll* was defined as ‘interrupting sitting time,’ changing rooms in facilities or walking to group activities counted as successful *Stroll*.

### The influence of resident PA cooperation

Cooperation with institutions, especially in neighbourhoods, like preschools or nursery schools, were a popular option for integrating external activity promoters. Due to staff shortages, many actions developed within the participatory integrated counselling approach were group-oriented and offered by volunteers to activate as many residents as possible. Volunteers, in particular, took on a variety of PA-promoting actions, such as *Bowling* or *Musical Offers*—it guaranteed regular implementation even with staff shortages and additionally covered the social component [2, 43]. However, bureaucratic hurdles, such as mandatory contracts or fear of missing legal liabilities, often hindered establishing such cooperation, especially in urban areas. As a result, it was not always possible to offer the desired variety of activities since staff were often occupied with basic care. Here, confirmed Baert et al.‘s findings: the extent of PA depends mostly on staff’s capacity and also on personal attitudes towards PA since the remaining time after basic care can often be arranged by staff according to their preferences. In peripheral and urban areas, actions offered by external activity promoters were sometimes rated worse than expected (e.g., *PA Patrons*) due to more time-consuming planning processes; therefore, actions could only be initiated with enormous delays. However, to minimise reliance on external activity promoters, project nursing homes also provided targeted training for staff. Additionally, promoting PA was included in education curricula [7, 43]; it ensured adequate numbers of PA offerings not solely dependent on external activity promoters and minimised fears of failure promoting PA among staff [2, 7].

### Strengths and limitations

Our study strengths include involving staff and residents in the various workshops; all 7 nursing homes developed actions specifically tailored to their conditions. Since implementing PA increased from providing offers for the maintenance and promotion of mobility [41], our study supported nursing homes meeting recent mandatory care standards for maintaining mobility (§ 113a SCGB IX). Furthermore, financial resources for PA promotion were partly provided by the project, which made successful implementation of actions much more likely.

Study limitations include constant personnel changes (home management, nursing staff), which delayed project progress. In addition to staff changes, the pandemic also contributed to delayed schedules as time frames were unmet and evaluation workshops occurred 1 year after future workshop II. Overall, working with people in nursing home settings allowed insights into real organisational structures and PA-promoting action conditions as developed and implemented.

## Conclusion

The multitude of influencing factors, such as structural conditions, peripheral or urban locations, number of volunteers, and missions and visions of carriers requires home-specific actions for promoting PA. Only offering predetermined and time-limited interventions is insufficient. PA-promoting actions can be developed and integrated within a participatory integrated counselling approach with different stakeholders. Instead of a scientific project team, other activity experts (e.g., physiotherapists) can also guide counsellings as the specifications are sufficiently standardised [44]. Low-threshold opportunities usually hold promise for promoting PA successfully and either directly or indirectly related to PA. Although partner cooperations in the field are time-intensive to set up, they are often long-lasting. The most effective actions occurred in the area of neighbourhood, green care, individual activity behaviour, and specification of the nursing home. Actions such as strolls (neighbourhood) or raised flower beds (green care) reflect the former life of the residents and are a familiar pastime. Actions such as household activities (individual activity behaviour) can be highly individualised, and residents feel as a part of everyday duties. Specifications of the nursing home build the base of successful PA promotion by providing budget, material, and personnel. Future research should focus on processes of change at structural levels to better understand the complex phenomena of promoting PA in nursing home settings. Nevertheless, every individual and organisational step counts when it comes to PA promotion among nursing home residents.

## Data Availability

No datasets were generated or analysed during the current study.

## Abbreviations

PA: Physical Activity
GAS: Goal Attainment Scaling
WHO: World Health Organization
